# Shifts in uterine microbiome associated with pregnancy outcomes at first insemination and clinical cure in dairy cows with metritis

**DOI:** 10.1038/s41598-024-61704-0

**Published:** 2024-05-24

**Authors:** Caio C. Figueiredo, Hugo F. Monteiro, Federico Cunha, Danilo Z. Bisinotto, Angel Revilla Ruiz, Gustavo A. Duarte, Yong Ge, Fábio S. Lima, Mansour Mohamadzadeh, Klibs N. Galvão, Rafael S. Bisinotto

**Affiliations:** 1 Department of Large Animal Clinical Sciences, D. H. Barron Reproductive and Perinatal Biology Research Program, University of Florida, Gainesville, 32610 USA; 2 Department of Veterinary Clinical Sciences, Washington State University, Pullman, 99164 USA; 3 Department of Population Health and Reproduction, University of California, Davis, 95616 USA; 4 Department of Animal Sciences, University of Florida, Gainesville, 32611 USA; 5 Department of Microbiology, Immunology and Molecular Genetics, University of Texas Health, San Antonio, 78229 USA

**Keywords:** Bacteria, Clinical microbiology, Microbiology, Diseases

## Abstract

Objectives were to assess differences in uterine microbiome associated with clinical cure and pregnancy outcomes in dairy cows treated for metritis. Cows with metritis (reddish-brownish, watery, and fetid vaginal discharge) were paired with cows without metritis based on parity and days postpartum. Uterine contents were collected through transcervical lavage at diagnosis, five days later following antimicrobial therapy (day 5), and at 40 days postpartum. Uterine microbiome was assessed by sequencing the V4 hypervariable region of the 16S rRNA gene. Although alpha-diversity based on Chao1, Shannon, and inverse Simpson indexes at diagnosis did not differ between cows with and without metritis, disease was associated with differences in beta-diversity. Prevalence of *Porphyromonas*, *Bacteroides*, and *Veillonella* was greater in cows with metritis. *Streptococcus*, *Sphingomonas*, and *Ureaplasma* were more prevalent in cows without metritis. Differences in beta-diversity between cows with and without metritis persisted on day 5. Uterine microbiome was not associated with clinical cure. Richness and alpha-diversity, but not beta-diversity, of uterine microbiome 40 days postpartum were associated with metritis and pregnancy. No relationship between uterine microbiome and pregnancy outcomes was observed. Results indicate that factors other than changes in intrauterine bacterial community underlie fertility loss and clinical cure in cows with metritis.

## Introduction

Metritis is an inflammatory uterine disease of polymicrobial origin that affects approximately 25% of dairy cows within the first three weeks postpartum^1^. Besides representing a major welfare concern associated with pain and behavioral changes^2,3^, metritis is associated with reduced milk production, impaired reproductive efficiency, and increased risk of culling and death^4–7^. Altogether, the economic burden of metritis to the USA dairy industry has been estimated at nearly US$ 650 million/year^8^. Fertility loss observed in cows diagnosed with metritis is likely mediated by a combination of increased incidence of subsequent uterine diseases (e.g., purulent vaginal discharge and cytological endometritis), delayed resumption of ovulation postpartum, impaired fertilization following insemination, increased early embryonic mortality, and greater incidence of fetal losses compared with counterparts without metritis^5,9,10^. Loss of fertility, however, does not affect all cows with metritis as approximately 30% of them are able to establish and maintain pregnancy following the first insemination postpartum^5,9,11^. These data suggest that, amidst several biological processes that contribute to the clinical presentation of the disease, underlying mechanisms specifically linked to fertility loss exist and are not present in every cow diagnosed with metritis.

Mounting evidence indicate that differences in the structure and function of bacterial communities within the uterus are linked to resolution of uterine diseases and fertility responses in dairy cows. For instance, dairy cows diagnosed with purulent vaginal discharge in the presence of *Trueperella pyogenes* had lesser pregnancy per artificial insemination (AI) and longer interval from calving to establishment of pregnancy compared with cows with purulent vaginal discharge in the absence of this pathogen^12^. The presence of *Lactobacillus *spp. in the uterus of postpartum dairy cows, on the other hand, was associated with a reduced proportion of polymorphonuclear cells in endometrial cytology^13^, suggesting a possible role for *Lactobacillus *spp. in reducing uterine inflammation. In fact, postpartum intrauterine infusion of *Lactobacillus buchneri* in dairy cows with cytological endometritis reduced endometrial mRNA abundance of transcripts for several inflammatory cytokines, improved pregnancy per AI following first insemination postpartum, and reduced time to pregnancy^14^. The clinical cure within ten days of metritis diagnosis and onset of antimicrobial therapy has been associated with subsequent uterine health and fertility outcomes in lactating dairy cows^5^. Changes in the composition of the uterine microbiome have also been associated with clinical cure failure in dairy cows treated for metritis. Cows that failed to achieve clinical cure had greater abundance of bacteria from the genera *Bacteroides*, *Porphyromonas*, and *Fusobacterium* compared with cows that underwent clinical cure in response to antimicrobial therapy^15^.

The main hypothesis of the current study was that the uterine microbiome in dairy cows with metritis that become pregnant following the first AI postpartum differs from that of cows with metritis that fail to become pregnant. Moreover, we hypothesized that the microbiome of cows with metritis that become pregnant following the first AI postpartum is similar to that observed in cows without metritis independent of pregnancy outcomes. Finally, we hypothesized that clinical cure failure in dairy cows treated for metritis is associated with differences in the uterine microbiome. Therefore, the objectives were to characterize the uterine microbiome in dairy cows with and without metritis on the day of the diagnosis, five days later upon completion of antimicrobial therapy, and at 40 days postpartum.

## Results

Five samples were not included in statistical analyses because no taxa were observed, including samples from two cows with metritis and two cows without metritis collected at 40 days postpartum, and one sample from a cow without metritis collected on day 5. A total of 7,378 unique ASV were identified after the taxonomy assignment using the *dada2* pipeline processing. All supplementary figures and tables are available at 10.6084/m9.figshare.24029880. The mean (± standard deviation) days postpartum at first artificial insemination was 79.6 (± 4.9) for No metritis pregnant; 77.9 (± 4.9) for No metritis not pregnant; 80.0 (± 5.1) for Metritis pregnant; and 77.8 (± 5.1) for Metritis not pregnant. No major variation in days postpartum at first artificial insemination were observed among groups.

### Uterine microbiome according to metritis and pregnancy status

**Figure 1:**
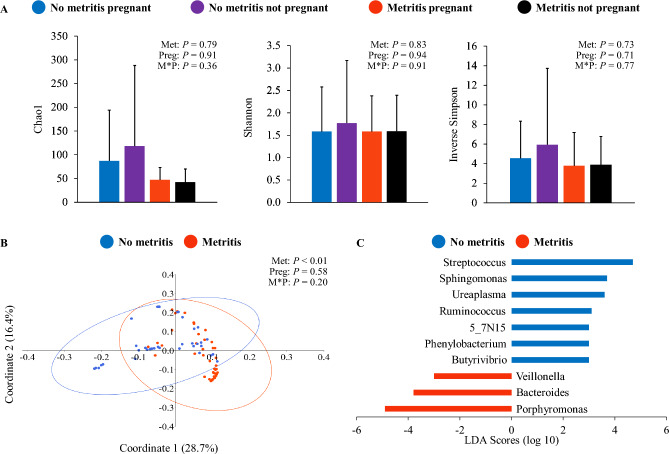
Uterine microbiome associated with metritis and pregnancy status following first artificial insemination postpartum on the day of metritis diagnosis (day 0). Panel A: evaluation of alpha-diversity according to metritis and pregnancy status following first artificial insemination postpartum based on richness and diversity indexes (mean ± SD). Panel B: principal coordinate analysis with Bray–Curtis dissimilarity (PERMANOVA) at the genus level for the main effect of metritis. Panel C: discriminant analysis for identification of differently abundant genera of bacteria between cows with and without metritis. Met = fixed effect of metritis; Preg = fixed effect of pregnancy status following first artificial insemination postpartum; M*P = interaction between the effects of metritis and pregnancy status following first artificial insemination postpartum.

Richness and diversity of the uterine microbial populations on day 0 were not associated (*P* ≥ 0.36) with metritis, pregnancy status following the first AI postpartum, or the interaction between the two variables (Fig. 1A). However, the uterine microbiome of cows with metritis differed (*P* < 0.0001) from that observed in cows without metritis on day 0 based on beta-diversity (Fig. 1B). Discriminant analysis revealed 16 genera of bacteria with an abundance that differed between cows with and without metritis, out of which 10 had been annotated (Fig. 1C). Conversely, shifts in uterine microbiome beta-diversity on day 0 were not associated with the main effect of pregnancy status following the first AI postpartum (*P* = 0.58) or the interaction between metritis and pregnancy status (*P* = 0.20). The results indicate that although 41 bacterial genera in the uterine microbiome were shared between cows with and without metritis on day 0, a large portion of microbial populations was exclusively observed in the uterus of cows without metritis (55 bacterial genera; Supplementary Figure S1A; Supplementary Table S1). Uterine lavage samples from cows with metritis had a reduced (*P* < 0.0001) number of bacterial DNA copies on day 0 compared with cows without metritis (metritis = 9.9 ± 2.9 vs. no metritis = 10.9 ± 4.7 log_10_ copies). Conversely, pregnancy status following the first AI postpartum (pregnant = 10.5 ± 4.2 vs. non-pregnant = 10.3 ± 3.5 log_10_ copies; *P* = 0.22) and the interaction between metritis and pregnancy status (*P* = 0.46) were not associated with the number of bacterial DNA copies on day 0.

**Figure 2:**
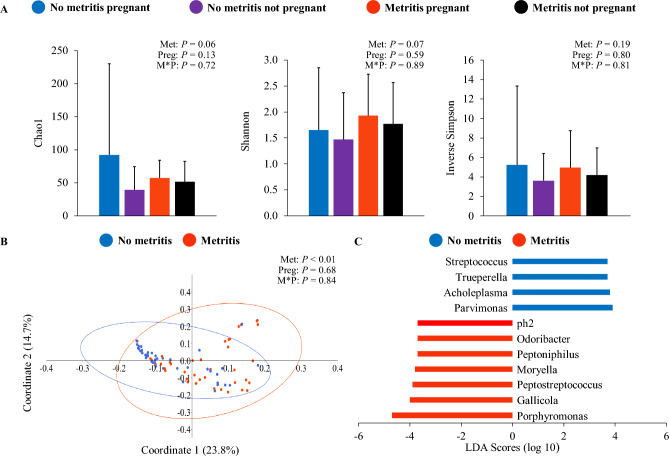
Uterine microbiome associated with metritis and pregnancy status following first artificial insemination postpartum on day 5. Panel A: evaluation of alpha-diversity according to metritis and pregnancy status following first artificial insemination postpartum based on richness and diversity indexes (mean ± SD). Panel B: principal coordinate analysis with Bray–Curtis dissimilarity (PERMANOVA) at the genus level for the main effect of metritis. Panel C: discriminant analysis for identification of differently abundant genera of bacteria between cows with and without metritis. Met = fixed effect of metritis; Preg = fixed effect of pregnancy status following first artificial insemination postpartum; M*P = interaction between the effects of metritis and pregnancy status following first artificial insemination postpartum.

Alpha-diversity of the uterine microbiome on day 5 was associated with metritis, as cows with metritis tended to have reduced richness according to Chao1 (metritis = 54.1 ± 29.2 vs. no metritis = 66.5 ± 104.2; *P* = 0.06) and tended to have greater diversity according to Shannon (metritis = 1.8 ± 0.8 vs. no metritis = 1.6 ± 1.0; *P* = 0.07) compared with cows without metritis (Fig. 2A). Pregnancy status after the first AI postpartum (*P* ≥ 0.13), or the interaction between metritis and pregnancy status (*P* ≥ 0.72) were not associated with the richness or diversity of the uterine microbiome. The uterine microbiome of cows with metritis on day 5 also differed (*P* = 0.0001) from that observed in cows without metritis based on beta-diversity (Fig. 2B). Discriminant analysis revealed 14 genera of bacteria with abundance that differed between cows with and without metritis, out of which 11 had been annotated (Fig. 2C). Shifts in uterine microbiome on day 5 were not associated with the main effect of pregnancy status following the first AI postpartum (*P* = 0.68) or the interaction between metritis and pregnancy status (*P* = 0.84). The results indicate that a large portion of microbial populations was exclusively observed in the uterus of cows without metritis at day 5, particularly in those that failed to achieve pregnancy following the first AI postpartum (69 bacterial genera; Supplementary Figure S1B; Supplementary Table S2). Uterine lavage samples from cows with metritis had reduced (*P* = 0.04) the number of bacterial DNA copies on day 5 compared with cows without metritis (metritis = 10.4 ± 2.5 vs. no metritis = 11.3 ± 2.3 log_10_ copies). Pregnancy status after the first AI postpartum tended to be associated with the number of bacterial DNA copies on day 5, as cows that became pregnant tended (*P* = 0.07) to have more bacterial DNA copies compared with non-pregnant cows (pregnant = 11.0 ± 2.8 vs. non-pregnant = 10.7 ± 2.0 log_10_ copies). The interaction between metritis and pregnancy status was not associated (*P* = 0.95) with the number of bacterial DNA copies in uterine lavage samples collected on day 5.

**Figure 3:**
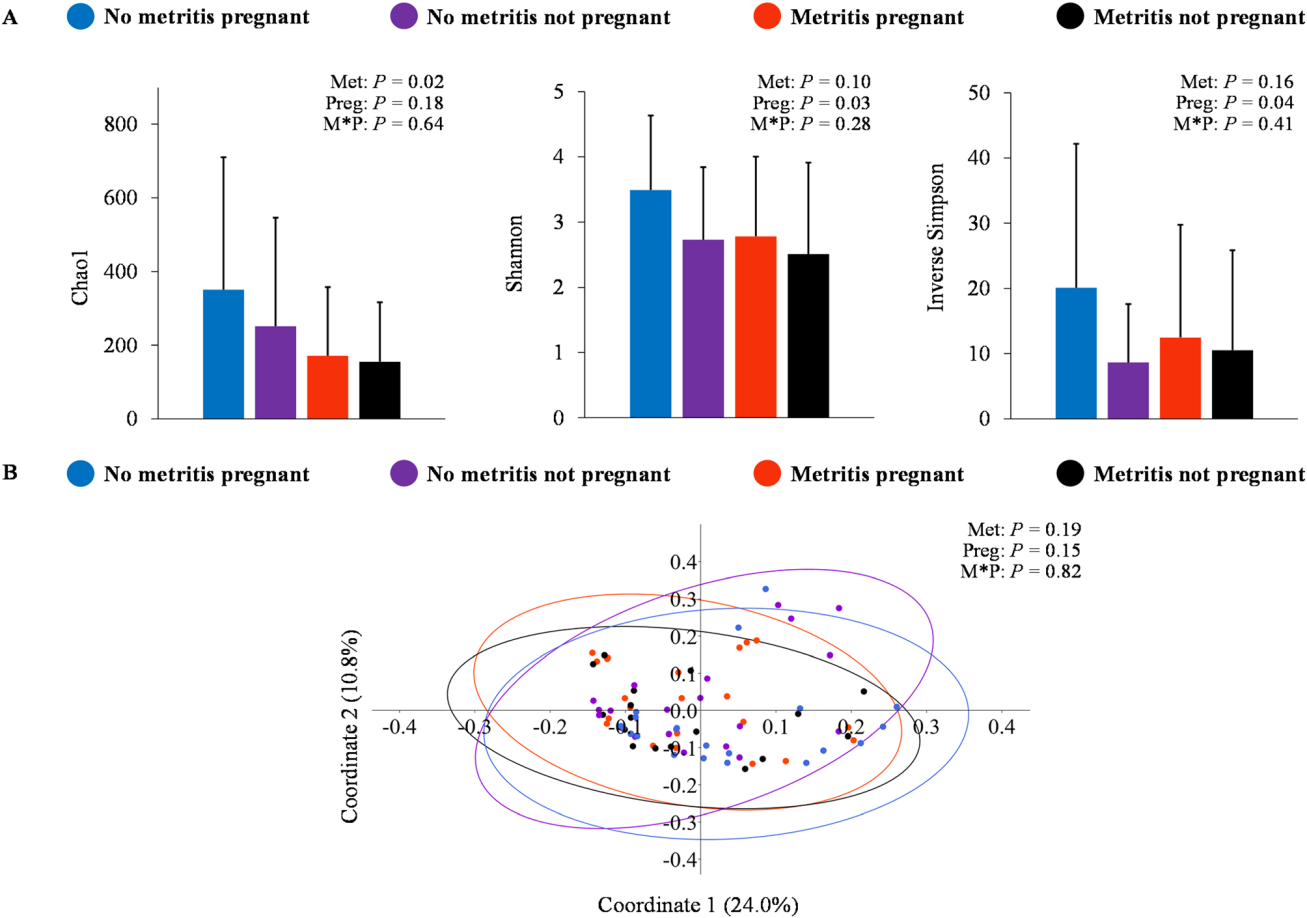
Uterine microbiome associated with metritis and pregnancy status following first artificial insemination postpartum at 40 days after calving. Panel A: evaluation of alpha-diversity according to metritis and pregnancy status following first artificial insemination postpartum based on richness and diversity indexes (mean ± SD). Panel B: principal coordinate analysis with Bray–Curtis dissimilarity (PERMANOVA) at the genus level for the main effects of metritis and pregnancy status following first artificial insemination postpartum. Met = fixed effect of metritis; Preg = fixed effect of pregnancy status following first artificial insemination postpartum; M*P = interaction between the effects of metritis and pregnancy status following first artificial insemination postpartum.

Similar to day 5, alpha-diversity of the uterine microbiome at 40 days postpartum was associated with metritis. Cows with metritis had reduced richness according to Chao1 (metritis = 161.9 ± 171.2 vs. no metritis = 304.1 ± 330.0; *P* = 0.02) and tended to have smaller diversity according to Shannon (metritis = 1.8 ± 0.8 vs. no metritis = 1.6 ± 1.0; *P* = 0.10) compared with cows without metritis (Fig. 3A). Moreover, cows that became pregnant after the first AI postpartum had greater diversity based on Shannon (pregnant = 3.2 ± 1.2 vs. non-pregnant = 2.6 ± 1.3 log_10_ copies; *P* = 0.03) and inverse Simpson indexes (pregnant = 16.6 ± 20.1 vs. non-pregnant = 9.7 ± 12.8 log_10_ copies; *P* = 0.04). The interaction between metritis and pregnancy status was not associated (*P* ≥ 0.28) with the richness or diversity of the uterine microbiome at 40 days postpartum. Despite differences in richness and diversity, uterine microbiome beta-diversity was not associated with metritis, pregnancy status after the first AI postpartum, or the interaction between metritis and pregnancy status (Fig. 3B). Venn-diagram revealed great homogeneity in the uterine microbiome composition among groups at 40 days postpartum, while few genera were observed exclusively for each group (Supplementary Figure S1C; Supplementary Table S3). The number of bacterial DNA copies at 40 days postpartum was not associated with metritis (metritis = 12.0 ± 3.7 vs. no metritis = 10.3 ± 5.7 log_10_ copies; *P* = 0.63), pregnancy status after first AI postpartum (pregnant = 10.5 ± 5.6 vs. non-pregnant = 11.7 ± 4.0 log_10_ copies; *P* = 0.87), or the interaction between metritis and pregnancy status (*P* = 0.24).

### Uterine microbiome according to clinical cure failure

**Figure 4:**
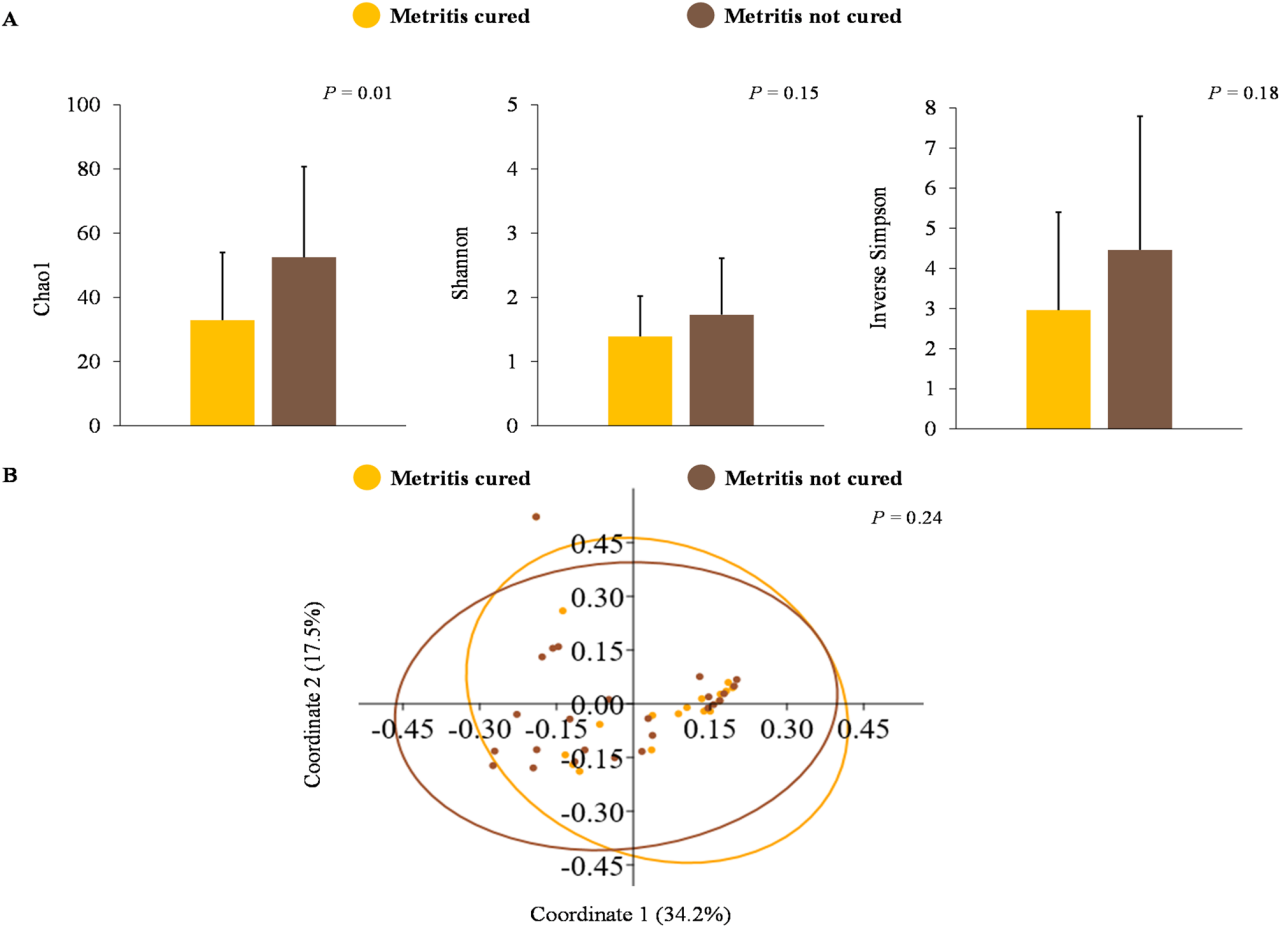
Uterine microbiome on the day of metritis diagnosis (day 0) associated with clinical cure failure. Panel A: evaluation of alpha-diversity according to clinical cure failure based on richness and diversity indexes (mean ± SD). Panel B: principal coordinate analysis with Bray–Curtis dissimilarity (PERMANOVA) at the genus level for the main effect clinical cure failure.

The richness of the uterine microbiome on day 0 based on the Chao1 index was greater (*P* = 0.01) for cows with clinical cure failure compared with cows that underwent clinical cure following antimicrobial treatment (Fig. 4A). Conversely, microbiome diversity based on Shannon and inverse Simpson indexes and beta-diversity did not differ (*P* ≥ 0.15) between cows with clinical cure failure and counterparts that underwent clinical cure (Figs. 4A, B). Venn-diagram revealed that although the majority of bacterial genera were observed in the uterus of cows with metritis on day 0 regardless of clinical cure status (37 genera), 22 bacterial genera were exclusively present in the uterine microbiome of cows with clinical cure failure (Supplementary Table S4). The number of bacterial DNA copies on day 0 did not differ (*P* = 0.59) between cows with and without clinical cure failure (cured = 9.9 ± 2.6 vs. not cured = 9.9 ± 3.1 log_10_ copies).

Microbiome richness based on Chao1 (cured = 47.3 ± 17.8 vs. not cured = 58.9 ± 34.8; *P* = 0.40), diversity based on Shannon (cured = 1.8 ± 0.7 vs. not cured = 1.9 ± 0.9; *P* = 0.68) and inverse Simpson (cured = 4.3 ± 2.7 vs. not cured = 4.7 ± 3.8; *P* = 0.84), and beta-diversity (*P* = 0.28) were not associated with clinical cure failure in cows treated for metritis. Venn-diagram revealed that the majority of bacterial genera were observed in the uterus of cows with metritis regardless of clinical cure status on day 5 (46 genera), and few were exclusively present in the uterine microbiome of cows with clinical cure failure (15 bacterial genera, Supplementary Table S5). The number of bacterial DNA copies in uterine lavage samples on day 5 (cured = 10.6 ± 2.9 vs. not cured = 10.3 ± 2.3 log_10_ copies; *P* = 0.27) was not associated with clinical cure failure in cows treated for metritis.

Similar to day 5, microbiome richness based on Chao1 (cured = 149.5 ± 206.0 vs. not cured = 170.7 ± 145.7; *P* = 0.18), diversity based on Shannon (cured = 2.5 ± 1.6 vs. not cured = 2.7 ± 1.1; *P* = 0.79) and inverse Simpson (cured = 15.6 ± 23.1 vs. not cured = 8.4 ± 7.2; *P* = 0.96), and beta-diversity (*P* = 0.87) did not differ between cows that cured following antimicrobial therapy and those with clinical cure failure. Venn-diagram revealed a large proportion of uterine microbial population was observed in the uterus of cows with metritis at 40 days postpartum regardless of clinical cure status, however, 55 bacterial genera were exclusively present in the uterus of cows with clinical cure failure (Supplementary Table S6). Number of bacterial DNA copies in uterine lavage samples at 40 days postpartum (cured = 11.8 ± 3.5 vs. not cured = 12.0 ± 3.9 log_10_ copies; *P* = 0.53) was not associated with clinical cure failure in cows treated for metritis.

## Discussion

Metritis has consistently been associated with reduced reproductive efficiency, through increased incidence of subsequent uterine diseases, delayed return to estrous cyclicity postpartum, reduced proportion of cows pregnant to first insemination, and increased time to pregnancy after parturition^5,9–11^. Nevertheless, approximately one in three cows diagnosed with metritis become pregnant following the first insemination postpartum^5,9–11^. These data support the hypothesis that fertility losses associated with metritis are explained by specific mechanisms that impair reproductive processes directly, or prevent the recovery of reproductive function postpartum, which are observed in a specific cohort within the population of cows with metritis at large. Contrary to the initial hypothesis, results from the present study indicate that the overall structure of the uterine microbiome at disease diagnosis, early after completion of antimicrobial therapy, and close to the time of insemination did not differ between cows with metritis that became pregnant to the first AI postpartum and cows with metritis that failed to become pregnant. Cows that became pregnant after the first AI postpartum had greater microbiome diversity close to the time of insemination (i.e., 40 days postpartum), which is contrary to data from beef cows subjected to timed AI^16^. Moreover, bacteria previously associated with decreased fertility responses in cows with uterine disease^11^ or increased reproductive performance^13,14^ were not associated with pregnancy status in the present study.

The pathogenicity of bacteria involved in uterine diseases is dependent on specific virulence factors, which have been associated with marked differences in disease incidence, tissue invasiveness, and subsequent fertility outcomes^17–19^. For instance, different clusters of *Escherichia coli* isolated from the uterus of dairy cows were characterized based on clinical signs and DNA polymorphism^17^. In vitro exposure of mice endometrial and stromal cells to *E. coli* from two out of four clusters resulted in increased tissue invasion. Presence of the virulence factors *fimH*, *astA*, *ibeA*, *cdt*, *hlyA* and *kpsMII* in *E. coli* isolates collected using uterine swabs were associated with increased incidence of metritis and purulent vaginal discharge in dairy cows^18^. Moreover, presence of *E. coli* positive for *fimH* was associated with smaller hazard of pregnancy in lactating dairy cows^19^. Differences in the prevalence of genes encoding for key virulence factors associated with fertility loss in cows with metritis could not be determined in the present study. Sequencing of the 16S rRNA gene is a useful approach to characterize the broad composition of the uterine microbiome to the level of genus. However, whole-genome shotgun sequencing, transcriptome analyses based on RNA sequencing, or targeting of specific virulence factors using PCR would have been needed to expand conclusions to the function of the uterine microbiome.

Another possible explanation for the absence of differences in uterine microbiome between cows with metritis that became pregnant to the first AI postpartum and cows with metritis that failed to become pregnant is related to the host response to infection. Intrauterine infusion of *E. coli* MS 499 (10 mL containing 4.64 × 10^7^ cfu/mL) and *T. pyogenes* MS249 (10 mL containing 3.38 × 10^7^ cfu/mL) following endometrial scarification and supplementation with progesterone was successful in inducing purulent vaginal discharge in non-lactating dairy heifers^20^. The rate at which heifers developed clinical signs varied among animals, thus, suggesting that resilience against the same source of infection was also variable. Using the same purulent vaginal discharge induction model, the transcriptome of granulosa cells, ampulla, isthmus, caruncular endometrium, and intercaruncular endometrium three months after bacterial infusion was compared between infected heifers and unchallenged controls^21^. Intrauterine bacterial infusion resulted in long-term changes in transcriptome, with mostly unique sets of differentially expressed transcripts in each tissue. Of particular importance for the discussion of present results, principal component analyses revealed considerable variability in within the group of heifers infused with bacteria, particularly regarding transcriptome changes in the granulosa cells. Cows classified with metritis based on vaginal discharge score postpartum (i.e., VDS = 5) have increased concentration of haptoglobin in serum compared with counterparts without metritis^2^. Interestingly, concentration of haptoglobin in serum and in vaginal discharge was positively correlated with vaginal discharge score^2,22^. Cows with metritis also displayed substantial variability in haptoglobin concentrations both in vaginal discharge and in serum^22^. Although the evaluation of intra-group variability was not one of the objectives from the studies described above and, therefore, formal analyses within group were not performed, these data support the hypothesis that cows respond differently to a similar insult.

Finally, differences in fertility within cows diagnosed with metritis can be caused by factors other than changes in the uterine microbiome. For instance, the degree of physical trauma secondary to parturition varies among cows with metritis. In a study wherein, lactating dairy cows were classified according to laceration at the dorsal commissure of the vulva or vaginal walls, approximately 18% of cows diagnosed with metritis had no signs of trauma whereas vulvovaginal lacerations up to 2 cm in length or greater than 2 cm were observed in 39 and 43% of cows with metritis, respectively^23^. Moreover, increasing vaginal laceration score was associated with delayed resumption of ovulation postpartum and reduced pregnancy per AI following the first insemination postpartum. Although uterine fluid was collected at similar days postpartum for all cows, the stage of estrous cycle was not evaluated or synchronized prior to collection. It represents a limitation of the present study as changes in hormonal profiling were previously associated with differences the uterine microbiome in cows.

Cows without metritis had a greater number of bacterial DNA copies in uterine lavage at the time of disease diagnosis compared with cows diagnosed with metritis in the present study. In addition, no differences in richness or diversity of the uterine microbiome were observed between cows with and without metritis on day 0. These results contradict previously published data in which cows with metritis were found to have greater number of bacterial DNA copies and reduced richness in uterine swab samples compared with cows without metritis at the time of diagnosis^24–26^. Such disparity in observations could be explained by differences in sampling methodology and experimental design.

Previous studies evaluated differences between cows with and without metritis independently of outcomes subsequent to disease diagnosis or shortly after completion of antimicrobial therapy. In the present study, on the other hand, cows were considered eligible for microbiome analyses only if the outcome of the first AI postpartum was known. Metritis is linked to increased mortality and culling during early lactation and to a decrease in the proportion of cows that receive a first AI postpartum^4,5,7^. Therefore, it is likely that restricting the cohort of cows with metritis to those deemed eligible for reproduction by farm personnel and that remained in the herd sufficiently long to undergo pregnancy diagnosis following the first AI postpartum yielded a different population compared with previous studies. It is important to highlight that such inclusion criteria were necessary for evaluation of the hypotheses proposed herein, and that a broad comparison between cows with and without metritis independently of fertility outcomes to match previous studies was not an objective of the present study. Despite differences in experimental design, changes in the uterine microbiome at the genus level observed between cows with and without metritis at the time of diagnosis in the present study agree with previously published data. For instance, prevalence of bacteria from the genus *Porphyromonas* and *Bacteroides* was greater in the uterus of cows with metritis compared with counterparts without metritis at the time of diagnosis, whereas greater prevalence of *Streptococcus*, *Ruminococcus*, and *Ureaplasma* was observed in cows without metritis^25^.

Jeon et al.^15^ showed greater prevalence of bacteria from the genus *Bacteroides*, *Porphyromonas*, and *Fusobacterium* in the uterus of cows with clinical cure failure compared with cows that cured following antimicrobial therapy. Except for greater richness on day 0 based on Chao1 in cows with clinical cure failure, no differences in uterine microbiome were observed between cows with metritis that cured and counterparts that failed to cure after treatment. Considering the similarity in the uterine microbiome of cows with metritis that cured and those with clinical cure failure on day 5, it is possible that other mechanisms related to immune response play a major role in clinical cure. Studies have reported that cows diagnosed with metritis and pyrexia (rectal temperature ≥ 39.5 °C) were less likely to cure compared with cows with metritis without pyrexia^5,27^. Furthermore, concurrent injuries and inflammation were associated with reduced a risk of achieving clinical cure. For instance, the presence of vulvovaginal laceration, and greater plasma concentration of haptoglobin at the time of metritis diagnosis were associated with reduced odds of achieving clinical cure in cows with metritis^28^. Sheldon et al.^29^ proposed a mechanistic model related to postpartum uterine disease prevention that consists of avoidance, tolerating, and resisting bacteria in the uterus. It was proposed that cows that are more efficient at limiting the exposure to bacteria (avoidance), limiting the damage caused by bacteria (tolerance), and limiting the number of bacteria (resistance) are, ideally, less likely to develop uterine diseases. It is possible that such mechanisms also play a role in clinical cure failure. More studies are warranted to explore changes in such mechanisms in cows with clinical cure failure, particularly allowing for the enrollment of cows with clinical cure failure that includes cows classified as reproductive culls and those that leave the herd before assessment of pregnancy status following the first AI postpartum.

## Conclusion

In conclusion, this study provides evidence that the reduction in fertility observed in cows with metritis compared with cows without metritis is not mediated by key differences in uterine microbiome. Moreover, clinical cure failure in cows treated for metritis was not associated with changes in uterine microbiome in the present study. Future research focusing on factors related to the function of uterine microbiome in cows with metritis and on risk factors for impaired fertility and clinical cure failure that are not related to uterine microbial populations is warranted.

## Materials and methods

All procedures involving animals were approved by the animal care and use committee of the University of Florida (IACUC, protocol no. 201810204). Authors confirm that the study reported here was conducted in accordance with relevant guidelines and regulations governing the use of experimental animals. This study has been reported in accordance with ARRIVE guidelines (https://arriveguidelines.org/; accessed 08/22/2023).

### Study population, housing, and reproductive management

This case-controlled study was conducted from February to November 2018 in two commercial dairy farms located in Florida, USA. The average number of lactating cows and rolling herd average for each farm were approximately 5,270 cows and 11,000 kg (farm 1) and 2,500 cows and 12,050 kg (farm 2). Lactating Holstein cows were milked thrice daily and fed twice daily a total mixed ration to meet or exceed the nutritional requirements of a 650 kg cow producing 40 kg/day of 3.5% fat-corrected milk^30^. In both farms, cows were housed in naturally ventilated free-stall barns equipped with sprinklers and fans over the feed bunk and fans over deep-bedded sand stalls. All procedures were discussed with owners and farm personnel, who consented to the use of their animals and facilities.

In farm 1, cows became eligible to receive AI after 60 days postpartum. Cows were treated with prostaglandin F_2α_ (PGF_2α_; 25 mg of dinoprost tromethamine i.m., Lutalyse, Zoetis, Madison, NJ, USA) at 50 ± 3 and 64 ± 3 days postpartum, and those detected in estrus based on removal of tail chalk received AI on the same day. Cows not inseminated by 76 ± 3 days postpartum were enrolled in a protocol for synchronization of ovulation and timed AI based on GnRH (100 μg of gonadorelin diacetate tetrahydrate, i.m., Cystorelin, Boehringer Ingelheim Vetmedica, St. Joseph, MO, USA) and PGF_2α_ (GnRH, 5 and 6 days later PGF_2α_, 2 days later GnRH and timed AI). Pregnancy was diagnosed at 34 ± 3 days after AI by transrectal ultrasonography. In farm 2, cows became eligible to receive AI after 76 days postpartum. All cows were enrolled in a protocol for synchronization of ovulation and timed AI starting at 39 ± 3 days postpartum (PGF_2α_, 14 days later PGF_2α_, 10 days later GnRH, 7 days later PGF_2α_, 3 days later GnRH and timed AI). Pregnancy was diagnosed at 40 ± 3 days after AI by rectal palpation.

### Metritis definition, and antimicrobial treatment

Metritis was diagnosed based on visual evaluation of vaginal discharge at 5, 7, and 11 days postpartum (farm 1) or 4, 6, 8, 10, and 12 days postpartum (farm 2) using a Metricheck device (Simcro, Hamilton, New Zealand). Vaginal discharge score (VDS) was evaluated using a 5-point scale (1 = clear mucus or lochia, 2 = clear mucus with flecks of pus; 3 = mucopurulent discharge with < 50% of pus; 4 = mucopurulent discharge with ≥ 50% of pus or reddish mucous discharge not fetid; 5 = watery, reddish or brownish, and fetid discharge) described for dairy cows^31^. Metritis was defined for cows with VDS = 5 (*n* = 43; farm 1, *n* = 30; farm 2, *n* = 13). Cows with metritis were paired with cows without metritis (VDS ≤ 3) based on parity (primiparous vs. multiparous) and calving date (*n* = 42; farm 1, *n* = 30; farm 2, *n* = 12), which were considered as negative controls. The day of metritis diagnosis and pairing of cows without metritis was considered study day 0.

All cows diagnosed with metritis received systemic antimicrobial therapy starting on day 0. Cows were treated either with 6.6 mg/kg of bodyweight of ceftiofur crystalline free acid s.c. (Excede Sterile Suspension, Zoetis, Madison, NJ, USA) injected twice 72 h apart (*n* = 38; farm 1, *n* = 30; farm 2, *n* = 8) or 11 mg/kg of bodyweight of ampicillin trihydrate i.m. (Polyflex, Boehringer Ingelheim Vetmedica) injected once daily during five consecutive days (farm 2, n = 5). Vaginal discharge was evaluated on day 5 for assessment of clinical cure in cows treated for metritis. Clinical cure following antimicrobial therapy was defined for cows with VDS ≤ 4 on day 5 (metritis cured; *n* = 18; farm 1, *n* = 12; farm 2, *n* = 6), whereas clinical cure failure was defined for cows with VDS = 5 on day 5 (metritis not cured; *n* = 25; farm 1, *n* = 18, farm 2, *n* = 7). Cows were categorized according to metritis and pregnancy status after the first AI postpartum as metritis pregnant (*n* = 19), metritis not pregnant (*n* = 24), no metritis pregnant (*n* = 22), and no metritis not pregnant (*n* = 20).

### Characterization of uterine microbial populations

Before initiation of antimicrobial treatments on day 0, all cows were subjected to a low-volume uterine lavage performed by a single technician. Briefly, cows were palpated rectally for the stabilization of the cervix, the vulva was cleaned with paper towel and ethanol (70% vol./vol.), and a single-use plastic round-tip pipette (UterFlush pipettes, Van Beek, Orange City, IA, USA) was introduced into the vagina at a 45° angle and manipulated through the cervix. A total of 30 mL of sterile saline solution (0.9% sodium chloride irrigation, Baxter, Deerfield, IL, USA) was infused into the uterine lumen, more specifically in the body of the uterus, using a 60 mL syringe (Covidien, Mansfield, MA, USA). Uterine contents were homogenized, retrieved into the same 60 mL syringe, and transferred to a sterile 15 mL conical tube (VWR, Radnor, PA, USA). Uterine lavage was also performed on day 5 and at 40 days postpartum in all cows. Tubes were placed on ice immediately after collection and transported to the laboratory within 6 h of collection. Uterine lavage samples were aliquoted into 2 mL microcentrifuge tubes (Eppendorf, Enfield, CT, USA) and stored at -80 °C until assayed.

Genomic DNA was extracted using a protocol described previously for uterine samples collected from cows with and without metritis^15^. Microcentrifuge tubes containing uterine lavage samples were centrifuged (AccuSpin Microcentrifuge; Fisher Scientific, Hanover Park, IL, USA) at 13,000 × *g* for 3 min and the supernatant was discarded. The remaining pellet was resuspended in 200 µL of buffer ATL (QIAamp DNA Mini kit, Qiagen, Germantown, MD, USA), 5 µL of lysozyme (50 mg/mL; Thermo Scientific, Waltham, MA, USA), and 6 µL of mutanolysin (14.3 IU/µL; Sigma, Burlington, MA, USA) were added, samples were incubated at 37 °C for 1 h. After incubation, 10 µL of RNAase A (20 mg/mL; Invitrogen, Waltham, MA, USA) was added and samples were incubated at room temperature for 2 min. After incubation, 20 µL of protease K (QIAamp DNA Mini kit, Qiagen) was added and samples were incubated at 56 °C for 36 min. Lastly, 300 µL of absolute ethanol (Fisher Scientific) was added, samples were pipetted into spin columns, and the remaining steps were followed according to manufacturer’s instructions (QIAamp DNA Mini kit, Qiagen). The concentration and purity of genomic DNA were evaluated by optical density using a NanoDrop spectrophotometer (Thermo Scientific).

Amplification was performed for each individual sample by PCR using the platinum *Taq* DNA Polymerase High-Fidelity kit (Thermofisher, Waltham, MA, USA) according to the manufacturer’s instructions. The V4-V5 regions of the 16S rRNA gene were amplified by using barcoded dual-index primers as described previously^32^. The PCR conditions for the 16S rRNA gene consisted of an initial denaturing step of 94 °C for 3 min, followed by 35 amplification cycles (94 °C for 45 s, 50 °C for 1 min, and 72 °C for 90 s), and an elongation step at 72 °C for 10 min. Replicate amplicons were pooled and visualized by electrophoresis through 1.2% (wt./vol.) stained agarose gels (GelStar Nucleic Acid gel Stain; Lonza, Morristown, NJ, USA) before sequencing. Reactions with blank controls (i.e., no DNA added to the reaction mixture) were performed to evaluate contamination throughout sample processing. Gel was extracted using E.Z.N.A. gel extraction kit (Omega Bio-Tek, Norcross, Georgia, USA), and DNA was quantified using the Qubit double-stranded DNA high-sensitivity assay kit (Molecular Probes, Eugene, OR, USA). Amplicon aliquots were standardized to the same concentration and then pooled into one of one run according to individual barcode primers of the 16S rRNA gene. Final equimolar libraries were sequenced using the MiSeq reagent kit V2 for 300 cycles (read length of 300 bases, sequenced as single read) on the MiSeq platform (Illumina Inc., San Diego, CA, USA). Quantitative RT-PCR and calculation of bacterial DNA copy number were performed in duplicate using KAPA Library Quantification Kits (Roche, Pleasanton, CA, USA) and Bio-Rad CFX96 Real-Time System (Bio-Rad, Hercules, CA, USA) following according to the manufacturer’s instructions.

### Sample size, bioinformatics, and statistical analyses

Data from previously published work in which the uterine microbiome of dairy cows with different health status (i.e., no metritis and endometritis, metritis only, metritis followed by endometritis, and endometritis only) was assessed at different intervals postpartum (i.e., 1 to 3, 8 to 10, and 34 to 36 days postpartum) were used for determination of sample size in the current study^33^. In that study, four experimental units per group allowed for the detection changes in relative abundance of relevant taxa. We assumed that a tenfold larger sample size for the evaluation of uterine microbiome in cows with (*n* = 43) and without metritis (*n* = 42) and a fivefold larger sample size for the assessment of the interaction between metritis and pregnancy status (metritis pregnant, *n* = 22; metritis not pregnant, *n* = 21; no metritis pregnant, *n* = 19; no metritis not pregnant, *n* = 23) and clinical cure (metritis cured, *n* = 18; metritis not cured, *n* = 25) were sufficient to ensure adequate power in the present study. Cow was considered the experimental unit for statistical analyses.

The 16S rRNA gene sequences were demultiplexed using the Quantitative Insights Into Microbial Ecology (QIIME) pipeline^34^. Denoising of sequenced amplicons and community analyses were performed using the *dada2* pipeline^35^ in R Studio. Demultiplexed fastq files from forward and reverse readings were inspected, filtered, and trimmed based on their quality scores and error rates. Only reads with ≥ 440 bases of combined forward and reverse readings after quality filtering were used for community analyses. Amplicon sequence variant (ASV) tables were created, forward and reverse readings were merged, chimeras were removed, and taxonomy was assigned using the 16S rRNA gene Greengenes database^36^ with the phyloseq package^37^. Total ASV was then split into taxonomy levels, and the relative abundance of the ASV within each taxonomy level was calculated using the phyloseq package. Alpha-diversity indexes (i.e., Chao1 richness, Shannon diversity, and Inverse Simpson diversity) were calculated at the genus level using the microbiome and vegan packages^38,39^.

Alpha-diversity indexes and bacterial copy numbers were analyzed by non-parametric one-way ANOVA using the NPAR1WAY procedure of SAS version 9.4 (SAS Institute, Cary, NC, USA). Results were presented as unadjusted means ± standard deviation (SD) unless stated otherwise. Beta diversity was evaluated at the genus level using the PAST package version 4.09^40^. Principal coordinate analysis (PCoA) of the Bray–Curtis dissimilarity was used to compare groups within each time point (day 0, day 5, and 40 days postpartum). Changes associated with metritis, pregnancy status, the interaction between metritis and pregnancy status, and clinical cure failure were evaluated using one- or two-way PERMANOVA based on Bray–Curtis dissimilarity and 9,999 permutations. *P*-values for multiple comparisons on PERMNOVA were adjusted using the Bonferroni method to control study-wide error rates. Differences with *P* ≤ 0.05 were considered significant and those with 0.05 < *P* ≤ 0.10 were considered tendencies. Whenever PERMANOVA revealed differences with adjusted *P* ≤ 0.05, linear discriminant analysis (LDA) effect size (LEfSE) was used to determine differently abundant genera among groups^41^. Comparisons with *P* ≤ 0.05 for both Kruskall-Wallis and Wilcoxon tests, and logarithmic LDA scores ≥ 2 were considered significant. Changes in uterine microbiome at the genus level associated with metritis and pregnancy status, and clinical cure failure were further explored using Venn diagrams (Bioinformatics & Evolutionary Genomics; http://bioinformatics.psb.ugent.be/webtools/Venn/).

### Supplementary Information

Supplementary Information 1.

Supplementary Information 2.

Supplementary Information 3.

Supplementary Information 4.

Supplementary Information 5.

Supplementary Information 6.

Supplementary Information 7.

Supplementary Information 8.

Supplementary Information 9.

## Data Availability

All raw data are available at Microbial Genomes NCBI (submission ID: SUB13522155; BioProject ID: PRJNA983811).
