# Supplementary material for: Shifts in uterine microbiome associated with pregnancy outcomes at first insemination and clinical cure in dairy cows with metritis
Source: Sci Rep. 2024 May 24;14:11864. doi: 10.1038/s41598-024-61704-0 (PMC11126406; doi:10.1038/s41598-024-61704-0)
Supplement: Supplementary file 2 — Supplementary Information 2. [file 41598_2024_61704_MOESM2_ESM.docx]

Supplementary Figure S1. Panel A. Uterine microbiome at genus level according to metritis and pregnancy status following first artificial insemination postpartum on the day of metritis diagnosis; Panel B. Uterine microbiome at genus level according to metritis and pregnancy status following first artificial insemination postpartum on the day 5; Panel C. Uterine microbiome at genus level according to metritis and pregnancy status following first artificial insemination postpartum at 40 days postpartum.
