# Supplementary material for: Shifts in uterine microbiome associated with pregnancy outcomes at first insemination and clinical cure in dairy cows with metritis
Source: Sci Rep. 2024 May 24;14:11864. doi: 10.1038/s41598-024-61704-0 (PMC11126406; doi:10.1038/s41598-024-61704-0)
Supplement: Supplementary file 3 — Supplementary Information 3. [file 41598_2024_61704_MOESM3_ESM.docx]

Supplementary Table 1. Core genera shared between cows with and without metritis at the time of diagnosis (day 0), according to pregnancy outcomes following the first AI.

Supplementary Table 2. Core genera shared between cows with and without metritis five days after diagnosis (day 5), according to pregnancy outcomes following the first AI.

Supplementary Table 3. Core genera shared between cows with and without metritis at 40 days after calving, according to pregnancy outcomes following the first AI.

Supplementary Table 4. Core genera shared between cows with and without clinical cure failure at the time of metritis diagnosis (day 0).

Supplementary Table 5. Core genera shared between cows with and without clinical cure failure five days after metritis diagnosis (day 5).

Supplementary Table 6. Core genera shared between cows with and without clinical cure failure at 40 days after calving, according to pregnancy outcomes following the first AI.
