# Supplementary material for: Shifts in uterine microbiome associated with pregnancy outcomes at first insemination and clinical cure in dairy cows with metritis
Source: Sci Rep. 2024 May 24;14:11864. doi: 10.1038/s41598-024-61704-0 (PMC11126406; doi:10.1038/s41598-024-61704-0)
Supplement: Supplementary file 3 [file 41598_2024_61704_MOESM1_ESM.docx]

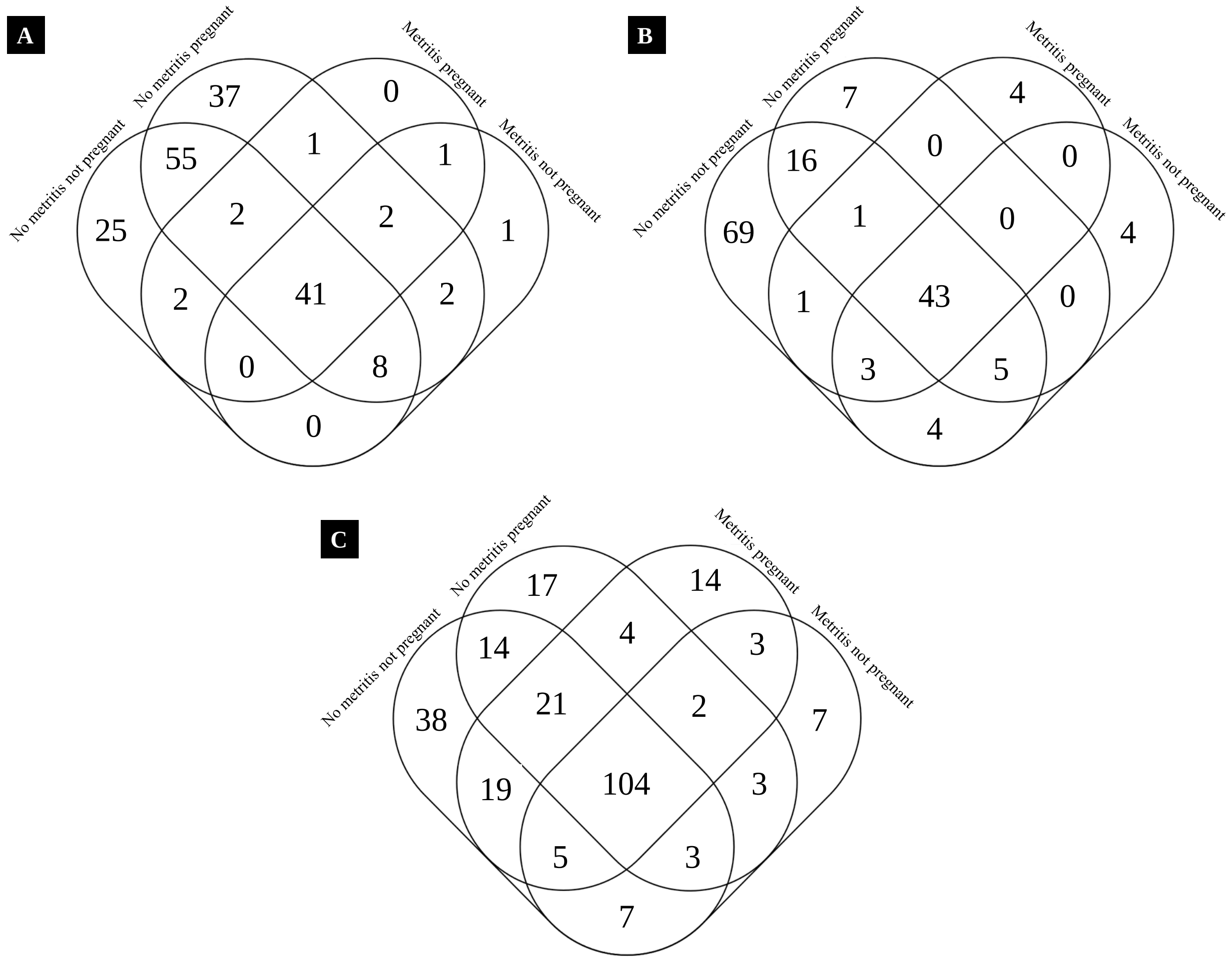
A
No metritis not pregnant
No metritis pregnant
Metritis pregnant
Metritis not pregnant
37
0
55
1
1
25
2
2
1
2
41
2
0
8
0
B
No metritis not pregnant
No metritis pregnant
Metritis pregnant
Metritis not pregnant
7
4
16
0
0
69
1
0
4
1
43
0
3
5
4
C
No metritis not pregnant
No metritis pregnant
Metritis pregnant
Metritis not pregnant
17
14
14
4
3
38
21
2
7
19
104
3
5
3
7
